# When the Body Hides the Ancestry: Phylogeny of Morphologically Modified Epizoic Earwigs Based on Molecular Evidence

**DOI:** 10.1371/journal.pone.0066900

**Published:** 2013-06-24

**Authors:** Petr Kocarek, Vaclav John, Pavel Hulva

**Affiliations:** 1 Department of Biology and Ecology, Faculty of Science, University of Ostrava, Ostrava, Czech Republic; 2 Department of Zoology, Faculty of Science, Charles University in Prague, Prague, Czech Republic; 3 Life Science Research Centre, Faculty of Science, University of Ostrava, Ostrava, Czech Republic; Université Claude Bernard - Lyon 1, France

## Abstract

Here, we present a study regarding the phylogenetic positions of two enigmatic earwig lineages whose unique phenotypic traits evolved in connection with ectoparasitic relationships with mammals. Extant earwigs (Dermaptera) have traditionally been divided into three suborders: the Hemimerina, Arixeniina, and Forficulina. While the Forficulina are typical, well-known, free-living earwigs, the Hemimerina and Arixeniina are unusual epizoic groups living on molossid bats (Arixeniina) or murid rodents (Hemimerina). The monophyly of both epizoic lineages is well established, but their relationship to the remainder of the Dermaptera is controversial because of their extremely modified morphology with paedomorphic features. We present phylogenetic analyses that include molecular data (18S and 28S ribosomal DNA and histone-3) for both Arixeniina and Hemimerina for the first time. This data set enabled us to apply a rigorous cladistics approach and to test competing hypotheses that were previously scattered in the literature. Our results demonstrate that Arixeniidae and Hemimeridae belong in the dermapteran suborder Neodermaptera, infraorder Epidermaptera, and superfamily Forficuloidea. The results support the sister group relationships of Arixeniidae+Chelisochidae and Hemimeridae+Forficulidae. This study demonstrates the potential for rapid and substantial macroevolutionary changes at the morphological level as related to adaptive evolution, in this case linked to the utilization of a novel trophic niche based on an epizoic life strategy. Our results also indicate that the evolutionary consequences of the transition to an ectoparazitic mode of living, which is extremely rare in earwigs, have biased previous morphology-based hypotheses regarding the phylogeny of this insect group.

## Introduction

The insect order Dermaptera (earwigs) comprises nearly 2000 extant described species [1], [2] in 11 families [3]. The monophyly of this group is well supported by morphological traits including the unsegmented forceps-like cerci of adults that assist in predation, mating, and wing folding [4], and also by the presence of holocentric chromosomes [5]. The Dermaptera had been traditionally divided into three suborders, the Hemimerina, Arixeniina, and Forficulina [see 5]–[9], and all three suborders were treated as monophyletic [3], [5], [10], [11]. But studies published during the last decades indicated that the Hemimeridae and Arixeniidae are highly specialized lineages within the traditional Forficulina [12]–[18].

According to the traditional concept of three suborders, the majority of earwig species were placed in the suborder Forficulina, which contains a wide variety of diverse lineages [19]. They are ‘typical earwigs’, i.e., free-living, feeding on plants or insects, oviparous or ovoviviparous [20], [21], and equipped with strong cerci. Some members of the Forficulina have complex wings and specialized wing-locking mechanisms on their tegmina, whereas others, like those in the Anisolabididae, are secondarily wingless [10]. According to the traditional concept, the Forficulina included nine families:: the Anisolabididae, Apachyidae, Chelisochidae, Diplatyidae, Forficulidae, Karschiellidae, Labiduridae, Pygidicranidae, and Spongiphoridae [3].

Hemimerids comprise 11 species, all commensal inhabitants of the fur of giant murid rats of the genera *Beamys* Thomas, 1909 and *Cricetomys* Waterhouse, 1840 in sub-Saharan Africa [22]. They are viviparous, which significantly increases the chances that the nymph finds the proper host. Synapomorphies supporting the monophyly of hemimerids are associated with adaptations to epizoic life. These include specialized grooves on the legs that allow the earwig to attach its legs close to the host body, loss of wings and eyes, and straight, narrow cerci [11], [22].

Arixeniids comprise five species occurring in Indonesia, the Philippines, and the Malay peninsula, and are associated with the molossid genus *Cheiromeles* Horsfield, 1824. Bats in this genus are characterized by their almost completely naked appearance, marked throat sack, and wing pouches that enable relatively advanced quadrupedal locomotion [22]. Arixeniid earwigs feed on skin and gland secretions of their hosts, but being frequently found also on guano in caves and trees, they are less closely associated with their hosts than are hemimerids. All arixeniids are viviparous. Synapomorphies include the absence of wings, reduced eyes, and the presence of thin, pubescent cerci that presumably serve a sensory function.

The systematic position of both epizoic lineages (the Hemimerina and Arixeniina) has been discussed throughout the history of their study. Walker [23], when he described the first *Hemimerus*, *H. taploides* Walker, 1871, placed this species in the Gryllidae. Saussure [24] erected the order Diploglossata to include *Hemimerus* and concluded that the new order should stand between Orthoptera and Thysanura. Sharp [25] even tried to place hemimerids among the Coleoptera near Platypsilidae, but three years later [26] created the family Hemimeridae, giving it equal rank in the orthopteroid stock to the Forficulidae, which at that time was used for all earwigs. Burr [27] considered the Hemimerina to represent a suborder of Dermaptera. This position was confirmed by many subsequent studies of external morphology [18], [21]–[30]. Popham [31] elevated the suprageneric status of Hemimerina to ordinal status, but Giles [8] challenged that change and reestablished the Hemimerina as a suborder within the Dermaptera.

When the genus *Arixenia* and the family Arixeniidae were described by Jordan [32], the author pointed out that if *Hemimerus* justified the creation of a separate suborder, a 3rd suborder of Dermaptera would have to be erected for the reception of the *Arixenia*. Some authors disagreed with Jordan, and Handlirsch [33] placed the Arixeniidae in the suborder Forficulina. Giles [8] considered the Arixeniina a suborder of Dermaptera and, based on external morphology, pointed out that 6 of 283 characters are common to the Forficulina and the Hemimerina but do not occur in the Arixeniina, that 12 characters are common to the Forficulina and Arixeniina but do not occur in the Hemimerina, and that the Arixeniina differ in 20 characters from the Hemimerina. However, these characters may be plesiomorphic, and the unique hemimerid and arixeniid characters may be independently derived as a result of an epizoic lifestyle. Alternatively, one might suspect that many of the features that make epizoic groups seem more primitive than Forficulina could be due to secondary reduction [12].

Popham [7], [34], [35] proposed a classification of the Dermaptera based on characters of the reproductive anatomy and primarily on the characters of the male external genitalia. Although he produced a large number of detailed drawings of genitalia, abdomens, and other structures, his proposed phylogeny was not based on a formal character analysis. Popham [7], [34], [35] considered arixeniids to be sister to the Spongiphoridae. Popham’s [7] hypothesis has not been generally accepted because it was based on an informal analysis rather than on a cladistic treatment of coded characters. Haas [10] performed the first quantitative phylogenetic analysis of the Dermaptera using morphological characters derived from 10 forficuline earwig taxa representing all nine recognized forficuline families and three blattid outgroups. Neither hemimerids nor arixeniids were included in his analysis. Haas & Kukalova-Peck [9] expanded on the Haas [10] matrix by including additional wing structure and venational characters and additional taxa, but hemimerids and arixeniids were again omitted. Klass [12] studied in detail the exoskeleton, musculature, and nervous system of the female abdomen of *Hemimerus vosseleri* Rehn & Rehn, 1935, and his results suggested that hemimerids are related to Apachyidae. The author [12] pointed out that hemimerids probably have many paedomorphic features in the thorax, in the female genital region, and in the cerci, which are thread-like.

Grimaldi & Engel [14] summarized information concerning the evolution of the Dermaptera mainly based on paleontological material. According to their concept, the Dermaptera consists of two primitive and extinct suborders, the Archidermaptera and Eodermaptera, and one recent suborder, the Neodermaptera. The latter is divided into the infraorder Protodermaptera, which contains the families Karscheiellidae, Diplatyidae, and Pygidicranidae, and the infraorder Epidermaptera, which contains the remaining eight families, including the Hemimeridae, nesting near the family Apachyidae, and the Arixeniidae, nesting near the Spongiphoridae (but the positions of the epizoic lineages are based on the poorly supported analyses of Popham [7], Haas & Kukalova-Peck [10], and Klass [12]). This classification was also adopted by Engel & Haas [15].

Karyotypic characters have not clarified the phylogeny of the suborders because the chromosome count (2n) ranges from 10 to 37 within the Forficulina [12], [36] and is 7 in the Arixeniina [37] and 60 in the Hemimerina [38].

Unfortunately, most of the recent systematic research on the Dermaptera has neglected the epizoic lineages altogether because fresh specimens for DNA analysis were unavailable [39]–[42]. Four molecular studies on Dermaptera have provided some additional insight into earwig phylogeny, but all lacked samples of hemimerids and arixeniids. Wirth et al. [39] sequenced 684 bp of cytochrome oxidase II across six dermapteran species. Guillet & Vancassel [40] reconstructed a phylogeny of 15 earwig species in four families based on l6S mitochondrial ribosomal sequence. Colgan et al. [41] included the widest selection of taxa and phylogenetic markers to date. Their analysis included 12 dermapteran taxa representing seven families and four outgroup taxa. They generated partial sequences from cytochrome oxidase I, 16S, 18S, and 28S. Kamimura [42] reconstructed an earwig phylogeny with representatives of seven families by using partial sequences of the mitochondrial 16S and nuclear 28S rRNA genes. The only study to consider the affinity of hemimerids in the phylogeny of the Dermaptera was that by Jarvis et al. [11], which was based on the sequences of the large ribosomal subunit 28S rRNA, small ribosomal subunit 18S rRNA, histone-3 (H3) nuclear DNA, and 43 morphological characters. The results indicated that the epizoic hemimerids are sister to Forficulidae+Chelisochidae but not to the remaining dermapterans.

Recently, Tworzydlo et al. [14], [16], [17] studied the ovary structure and initial stages of oogenesis in 15 representatives of several dermapteran taxa, including the epizoic *Arixenia esau* Jordan, 1909. The interpretation of the results in an evolutionary context supports the monophyly of the Dermaptera and the inclusion of the arixeniids in the clade Forficuloidea containing Spongiphoridae, Chelisochidae, and Forficulidae.

Here, we present a phylogenetic analysis of relationships between *Arixenia*, *Hemimerus*, and the remaining Dermaptera using 18S and 28S rRNA and histone-3 sequences. Our aim was to clarify the phylogeny of these morphologically modified epizoic earwigs and to establish their position within the Dermaptera based on molecular data.

## Materials and Methods

### Collecting of Samples

We used *Arixenia esau* Jordan, 1909 as representative of the Arixeniidae and *Hemimerus hanseni* Sharp, 1895 as representative of the Hemimeridae. *Arixenia esau* was collected in a cave in the Mulu area, Sarawak, Malaysia (3°57′N, 114°49′E) on 19 January 2009. The specimens were collected individually on rocks under the colony of the naked bulldog bat *Cheiromeles torquatus* Horsfield, 1824 and were preserved in pure ethanol. *Hemimerus hanseni* was collected in Big Babanki, Northwest Cameroon (6°7′N, 10°15′E) on 10 February 2007. The specimens were collected individually from the hairs of the Gambian pouched rat *Cricetomys gambianus* Waterhouse, 1840 that had been killed by automobiles and randomly found on the road. The material was preserved in 70% ethanol. *A. esau* and *H. hanseni* are not protected in the countries where they were collected, are not protected by any international law, and were not collected in protected areas. The taxa were unequivocally identified using the criteria in Nakata & Maa [15] and Rehn & Rehn [35]. The studied specimens are kept in the collection of Department of Biology and Ecology, University of Ostrava.

### Sequencing

Total genomic DNA was isolated from the femur of the leg with a Qiagen DNeasy Tissue kit (Qiagen) and following the manufacturer’s protocol. Three nuclear markers (18S and 28S ribosomal DNA and histone-3) were analyzed using primers published by Whiting [43] and Jarvis et al. [11] and listed in Table 1. PCR reactions were performed in 20-µl volumes containing 1×Taq buffer, 2.5 mM MgCl_2_, 200 µM dNTPs, 0.6 µM primers, 1 U Taq polymerase (Promega), and 50 ng of template DNA. The thermal protocol included predenaturation (95°C, 3 min); 10 cycles of denaturation (94°C, 60 s), annealing (54°C with a decrease of 0.5°C in each cycle, 90 s), and extension (72°C, 75 s); followed by 20 cycles identical to the previous 10 but with an annealing temperature of 48°C; and a final extension (72°C, 4 min). PCR products were examined on a 1% agarose gel, and amplified DNA was isolated from the gel using the QIAquick Gel Extraction Kit (Qiagen) or from a PCR mixture using the QIAquick PCR Purification kit (Qiagen). Purified PCR products were sequenced using the BigDye Terminator v3.1 Cycle Sequencing Kit (Invitrogen) and were analyzed on a 3130 Genetic Analyzer (Applied Biosystems). Results were viewed and edited using the program Chromas Lite v. 2.01 (http://www.technelysium.com.au/chromas_lite.html), and sequences were submitted to GenBank (Table 2).

**Table 1:** Primers used for amplification of 18S and 28S ribosomal DNA and histone-3 nuclear DNA (according to[11] and [42]).

Primer name	Sequence (5' → 3')
18S 1.2 F	TGCTTGTCTCAAAGATTAAGC
18S b5.0	TAACCGCAACAACTTTAAT
18S a0.7	ATTAAAGTTGTTGCGGTT
18S b0.5	GTTTCAGCTTTGCAACCAT
18S a2.0	ATGGTTGCAAAGCTGAAAC
18S 7R	GCATCACAGACCTGTTATTGC
18S 7F	GCAATAACAGGTCTGTGATGCCC
18S 9R	GATCCTTCCGCAGGTTCACCTAC
28S rD1.2a	CCCSSGTAATTTAAGCATATTA
28S Rd4.2b	CCTTGGTCCGTGTTTCAAGACGG
28S SA	GACCCGTCTTGAAGCACG
28S rD5b	CCACAGCGCCAGTTCTGCTTAC
28S Rd4.8a	ACCTATTCTCAAACTTTAAATGG
28S Rd6.2b	AATAKKAACCRGATTCCCTTTCGC
28S Rd6.2a	GAAAGGGAATCYGGTTMMTATTCC
28S rD7.b1	GACTTCCCTTACCTACAT
Hex AF	ATGGCTCGTACCAAGCAGACGGC
Hex AR	ATATCCTTGGGCATGATGGTGAC

**Table 2:** Information about specimens and GenBank accession numbers.

Family	Subfamily	Species	18s	28s	H3
Anisolabididae	Carcinophorinae	*Euborellia femoralis* (Dohrn, 1863)	AY707326, AY707349	AY707373, AY707393	AY707429
Anisolabididae	Carcinophorinae	*Thekalabis* sp.	AY707325, AY707348	AY707372, AY707392	AY707428
Anisolabididae	Parisolabiinae	*Parisopsalis spryi* Burr, 1914	-	AY144654	-
Apachyidae	Apachyinae	*Dendroiketes novaeguineae* Boeseman, 1954	AY521839	-	-
Arixeniidae	-	*Arixenia esau* Jordan, 1909	JX399774	JX399775	-
Chelisochidae	Chelisochinae	*Chelisoches morio* (Fabricius, 1775)	AY121133	AY125273	AY125220
Chelisochidae	Chelisochinae	*Chelisoches annulatus* Burr, 1906	AY707323, AY707346	AY707370, AY707390	AY707426
Chelisochidae	Chelisochinae	*Proreus duruoides* Hebard, 1933	AY707363	AY707384, AY707404	AY707439
Chelisochidae	-	-	AY521841	-	-
Forficulidae	Cosmiellinae	*Acanthocordax papuanus* Günther, 1929	AY707331, AY707354	AY707378, AY707398	-
Forficulidae	Ancistrogastrinae	*Ancistrogaster* sp.	AY144633	AY144660	-
Forficulidae	Forficulinae	*Doru spiculiferum* (Kirby, 1891)	AY121131	AY125272	AY125218
Forficulidae	Forficulinae	*Elaunon bipartitus* (Kirby, 1891)	AY707338, AY707361	AY707382, AY707402	AY707438
Forficulidae	Forficulinae	*Forficula* sp.	AY521836	AY521752	AY521703
Forficulidae	Forficulinae	*Forficula auricularia* Linnaeus, 1758	-	EU426876	-
Forficulidae	Opisthocosminae	*Eparchus biroi* (Burr, 1902) 1	AY707324, AY707347	AY707371, AY707391	AY707427
Forficulidae	Opisthocosminae	*Eparchus biroi* (Burr, 1902) 2	AY521837	AY521753, AY521754	-
Forficulidae	Opisthocosminae	*Opisthocosmia tenuis* Rehn, 1936	AY707321, AY707344	-	AY707425
Forficulidae	Opisthocosminae	*Paratimomenus* sp.	AY707322, AY707345	-	-
Hemimeridae	Hemimerinae	*Hemimerus* sp.	AY707334, AY707357	-	-
Hemimeridae	Hemimerinae	*Hemimerus hanseni* Sharp, 1895	JX399776	-	-
Labiduridae	Labidurinae	*Forcipula clavata* Liu, 1946	AY707320, AY707343	-	-
Labiduridae	Labidurinae	*Forcipula decolyi* de Bormans, 1900	AY707327, AY707350	AY707374, AY707394	AY707430
Labiduridae	Labidurinae	*Labidura riparia* (Pallas, 1773)	AY707333, AY707356	AY707380, AY707400	AY707435
Labiduridae	Nalinae	*Nala tenuicornis* (de Bormans, 1900)	AY707336, AY707359	-	AY707436
Labiduridae	Nalinae	*Nala lividipes* (Dufour, 1820)	AY707339, AY707362	-	-
Pygidicranidae	Echinosomatinae	*Echinosoma* sp.	AY121132	-	AY125219
Pygidicranidae	Echinosomatinae	*Echinosoma micropteryx* Günther, 1929	AY707330, AY707353	AY707377, AY707397	AY707433
Pygidicranidae	Echinosomatinae	*Echinosoma yorkense* Dohrn, 1869	AY144626	AY144652	-
Pygidicranidae	Pygidicraninae	*Cranopygia ophthalmica* (Dohrn, 1862)	AY707340, AY707364	AY707385, AY707405	AY707440
Pygidicranidae	Pygidicraninae	*Tagalina* sp.	AY521838	AY521756	AY521704
Spongiphoridae	Labiinae	*Labia* sp.	AY521840	AY521761, AY521762	-
Spongiphoridae	Nesogastrinae	*Nesogaster aculeatus* (de Bormans, 1900)	AY707335, AY707358	-	-
Spongiphoridae	Sparattinae	*Auchenomus forcipatus* Ramamurthi, 1967	AY707329, AY707352	AY707376, AY707396	AY707432
Spongiphoridae	Sparattinae	*Auchenomus* sp.	AY707337, AY707360	-	AY707437
Spongiphoridae	Spongiphorinae	*Irdex* sp. 1	AY707365	AY707386, AY707406	AY707441
Spongiphoridae	Spongiphorinae	*Irdex* sp. 2	AY707366	AY707387, AY707407	AY707442
					
	Family	Species	18s	28s	H3
Embioptera	Oligotomidae	*Oligotoma nigra* (Hagen, 1866)	AY121134	AY125274	AY125221
Embioptera	Teratembiidae	*Teratembia* sp.	AY121135	AY125275	AY125222
Grylloblattodea	Grylloblattidae	*Galloisana* sp.	AY707341, AY707367	AY707388, AY707408	AY707443
Grylloblattodea	Grylloblattidae	*Grylloblattina djakonovi* Bey-Bienko, 1951	AY707342, AY707368	AY707389, AY707409	AY707444
Orthoptera	Rhapidiophoridae	*Ceuthophilus utahensis* Thomas, 1876	AY521870	AY521800	AY521720
Orthoptera	Tridactylidae	*Ellipes minutus* (Scudder, 1892)	AY338723	AY338679	AY338641
Phasmida	Heteronemiidae	*Sceptrophasma langkawicensis* Brock & Seow-Choen, 2000	AY121166	AY125306	AY125249
Phasmida	Pseudophasmatidae	*Paraphasma rufipes* (Redtenbacher, 1906)	AY121160	AY125300	AY125244

### Assembling the Taxon-character Matrix

We used sequences from 35 other earwig species to determine the phylogenetic position of *Arixenia esau* and *Hemimerus hanseni* (Table 2). Among these comparative sequences, those from 34 species were obtained from Jarvis et al. [11], and those from one species were obtained from von Reumont et al. [44]. Following Jarvis et al. [11], we chose species from the orders Orthoptera, Phasmida, Embidiina, and Grylloblattodea as outgroups.

Because of large expansion regions in 28S and 18S ribosomal DNA and/or mutations in target sequences of universal primers for histone-3, the PCR amplification of some of these regions in the Dermaptera is problematic [cf. 11]. Sequences were aligned using Multalin [45] under default conditions and were edited in MEGA version 5 [46]. The use of different alignment software, e.g., Mafft [47], generated nearly identical results (data not shown). Regions with low consensus values (resulting from a lack of amplification, a lack of available complementary sequences, and/or a high degree of sequence polymorphisms including indels typical for non-protein coding regions) were excluded to avoid incorrect identification of homologies and/or overweighting of indels [48], [49]. Among the 18S DNA sequences, three regions with low consensus values were excluded: 48 bp at the 5′ end and 33 bp at the 3′ end were excluded because of a large number of missing sequences; a 156-bp region that included parts of the V4 hypervariable region described in De Rijk et al. [50] was excluded because of a combination of missing sequences and alignment failure. Among the 28S DNA sequences and following Jarvis et al. [11], we included a 509-bp region (including the D2 variable region described by De Rijk et al. [51]) at the proximal part (from the 5′ end) and a 378-bp region (including D4) in the middle of the gene, but we used more stringent conditions and therefore did not include the distal part of the gene because only a small number (<35%) of comparative sequences were available. A second alignment was derived from the variant described above by including only those parts with sequence information of *Arixenia* and *Hemimerus*. Both alignments are accessible at TreeBASE (http://purl.org/phylo/treebase/phylows/study/TB2:S14160).

### Phylogenetic Analyses

A basic description of the genetic variability within the data set was obtained using DnaSP v5 [52]. The data set was divided into three parts corresponding to 18S DNA, 28S DNA, and histone-3 sequences. For each part, the best model of sequence evolution was calculated with the aid of MrModelTest 2.3 [53] using Akaike Information Criterion (AIC).

Bayesian analyses were conducted in MrBayes 3.2.1 [54] using the resultant GTR+I+Γ model. Each analysis consisted of two independent runs for 40 million generations; trees were sampled every 5000 generations, and the first 25% were discarded as burn-in. The AWTY (Are We There Yet?) approach was used to explore the convergence of MCMC in Bayesian inference [55]. The stationarity of the runs was inspected by cumulative plots displaying the posterior probabilities of splits at selected increments over an MCMC run, and the convergence was visualized by comparative plots displaying posterior probabilities of all splits for paired MCMC runs.

## Results

**Figure 1:**
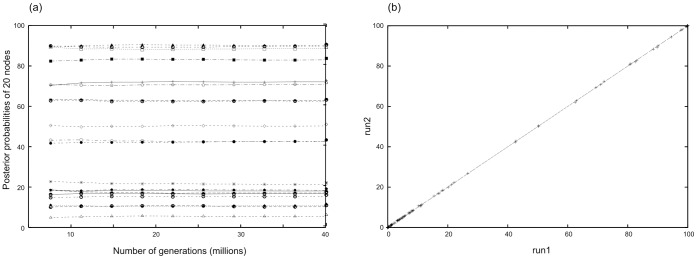
Results of the exploration of MCMC convergence using the AWTY (Are We There Yet?) approach. (a) Cumulative plot of the posterior probabilities of 20 splits at selected increments over one of two MCMC runs. (b) Comparative plot of posterior probabilities of all splits for paired one and two MCMC runs.

The resultant alignment contained 2876 characters, including 1665 bp from 18S, 835 bp from 28S, and 376 bp from H3; 1853 positions in total were variable. The shortened alignment contained 2365 characters, including 1585 bp from 18S and 780 bp from 28S; 1531 positions in total were variable. Based on AIC in MrModelTest and ModelTest analyses, the GTR+I+Γ model was chosen as the best in all three independent analyses for individual partitions in both alignments. Within Bayesian phylogenetic inference, two chains converged at similar topologies. The standard deviation of the split frequencies reached values lower than 0.01 during the analysis. The stationarity was reached after approximately 2×10^7^ generations (Fig. 1).

**Figure 2:**
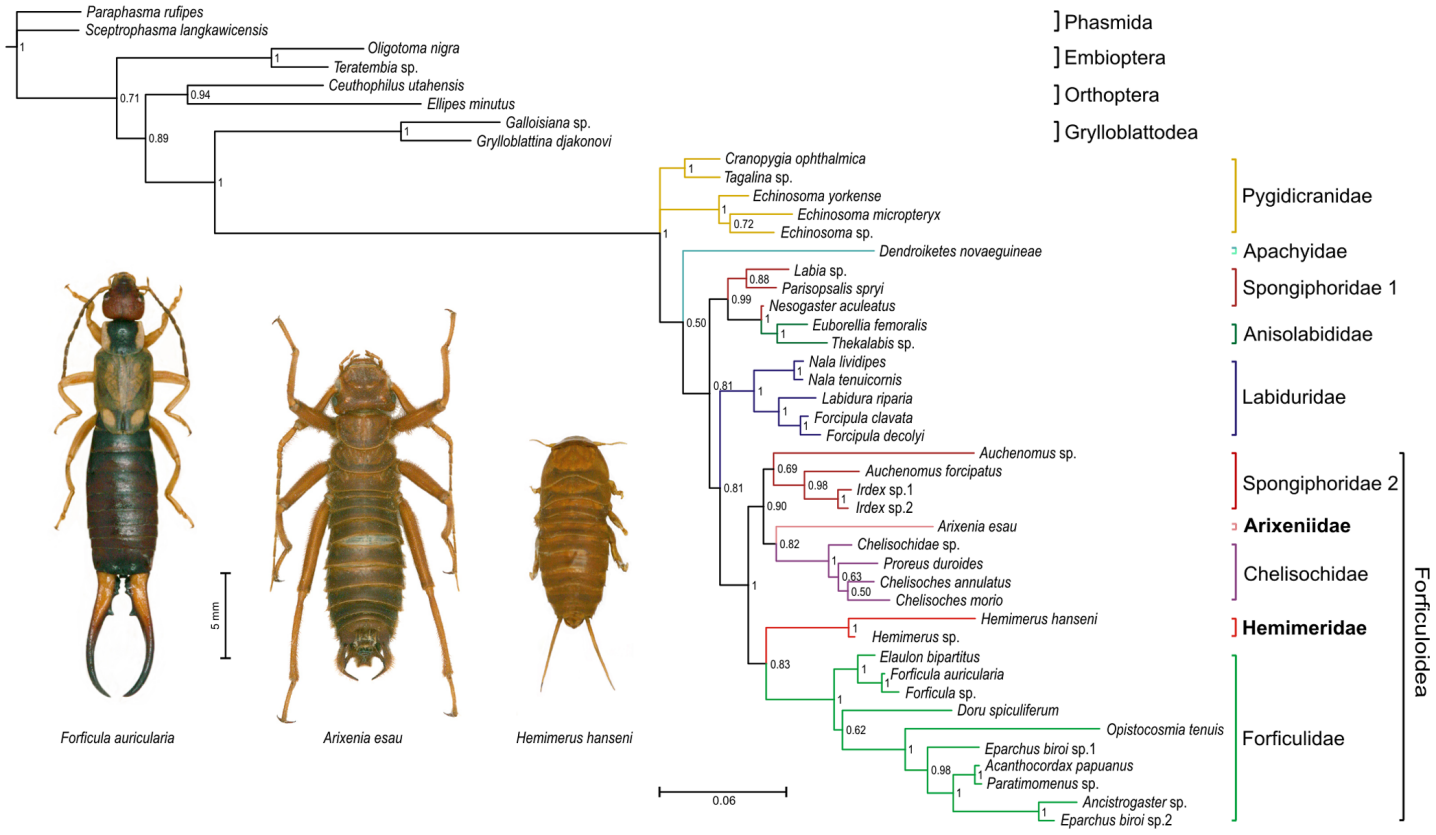
Bayesian phylogram of earwig families based on nuclear sequence data (18S and 28S ribosomal DNA and histone-3). Numbers above branches indicate posterior probabilities.

Bayesian analysis of the combined molecular data set produced a moderately resolved tree with several clear patterns (Fig. 2). Pygidicranidae, represented by *Cranopygia*, *Tagalina*, and *Echinosoma*, is unresloved in the tree with clades *Cranopygia*+*Tagalina* (PP(posterior probability) = 0.99) and *Echinosoma* (PP = 1) are monophyletic. Apachyidae is sister to the rest of the Dermaptera taxa except Pygidicranidae (PP = 0.50). Spongiphoridae is a polyphyletic/paraphyletic group with clades provisionally designated Sponiphoridae 1 and Spongiphoridae 2. The paraphyletic clade Spongiphoridae 1/Anisolabididae (PP = 0.99) contains the genera *Labia*, *Parisolabis*, *Nesogaster, Euborellia,* and *Thekalabis*. The monophyletic clade Spongiphoridae 2 (PP = 0.69) comprising *Auchenomus*+*Irdex* is sister to the Arixeniidae+Chelisochidae clade (PP = 0.82). Spongiphoridae 2+Arixeniidae+Chelisochidae+Hemimeridae+Forficulidae form a well-supported monophyletic clade Forficulidae (PP = 0.99), with two branches. Arixeniidae is sister to the Chelisochidae and forms a monophyletic clade with Spongiphoridae 2 (PP = 0.90), and Hemimeridae is sister to the monophyletic Forficulidae (PP = 0.83).

Bayesian analysis of the shortened alignment produced a moderately resolved tree similar to that produced by the combined molecular data set (Figure S1). Spongiphoridae 2+Arixeniidae+Chelisochidae+Hemimeridae+Forficulidae consistently form a well-supported monophyletic clade Forficulidae (PP = 0.99), with three polytomic branches: Spongiphoridae 2 (PP = 0.95), Arixeniidae+Chelisochidae (PP = 0.69), and Hemimeridae+Forficulidae (PP = 0.64). Arixeniidae is sister to the Chelisochidae and forms a monophyletic clade, and Hemimeridae is sister to the monophyletic Forficulidae.

## Discussion

### Molecular Phylogeny of Arixeniids and Hemimerids

The current study is the first to include molecular data for both arixeniids and hemimerids in one phylogenetic analysis. This data set enabled us to apply a rigorous cladistics approach and to test competing hypotheses that were previously scattered in the literature. Based on the results, we conclude that Arixeniidae and Hemimeridae are highly specialized and modified families belonging to the suborder Neodermaptera and infraorder Epidermaptera [11]–[18]. The results placed both groups in the superfamily Forficuloidea and support the sister group relationships of Arixeniidae+Chelisochidae and Hemimeridae+Forficulidae.

The genetic markers used in our study (18S and 28S ribosomal DNA and histone-3) are highly conserved among animals and have been traditionally used in phylogenetic studies [56], [57]. However, complete resolution of the deep branching in the order Dermaptera, which originated in the middle Mesozoic [14], will require analysis of long portions of nuclear DNA. Given the moderate values of posterior probabilities accompanying the nodes for Arixeniidae+Chelisochidae and Hemimeridae+Forficulidae, analysis of long portions of nuclear DNA will be also important for fine-scale resolution of the branching order within the superfamily Forficuloidea. The latter kind of phylogenomic analysis was required, for example, for the fine-scale resolution of mammalian and avian evolutionary histories [58], [59]. Nevertheless, the methods and tests used in our study indicate that our hypotheses regarding the phylogenetic position of groups of our interest, the Arixeniidae and Hemimeridae, are well supported. Our report is the first to use the Bayesian tree-building approach to resolve dermapteran phylogeny. This method is characterized by high power and consistency and is especially useful in crossing deep valleys in a virtual landscape of phylogenetic trees, thereby ensuring an increased probability of reaching global and not only local optima [60]. Exploration of MCMC using AWTY indicates that stationarity and convergence of the chains were reached during particular runs.

The only previous molecular study that included hemimerids [11] indicated that the group nested within the Neodermaptera, but the exact placement was poorly supported. That analysis, which treated parameter values as unity, placed *Hemimerus* as sister to Forficulidae+Chelisochidae but with relatively low bootstrap and Bremer support. Our study, in contrast, well supports the position of both epizoic Hemimeridae and Arixeniidae in the monophyletic group Forficuloidea representing part of Spongiphoridae +Arixeniidae+Chelisochidae+Hemimeridae+Forficulidae; the Hemimeridae is clearly sister to the monophyletic Forficulidae.

Our results suggest (as do the results of Jarvis et al. [11]) that the Spongiphoridae is not a monophyletic group but rather consists of two distinct lineages. One lineage is paraphyletic and contains the entire Anisolabididae clade, and other lineage is nested in the “Eudermaptera” and is sister to the Chelisochidae. Discussions of relationships within the Dermaptera based on morphological characters have generally focused on the male genitalia and have subdivided the Dermaptera into two infraorders, the Catadermaptera (with two penis lobes) and the Eudermaptera (with one lobe) [61]–[64]. Colgan et al. [41] demonstrated that division of Dermaptera into the “Catadermaptera” and “Eudermaptera” is not supported because the former seems to be paraphyletic. “Eudermaptera” was considered to be monophyletic by these authors. However, polyphyly of Spongiphoridae, which was indicated by Jarvis et al. [11] and was confirmed in this study, is inconsistent with Steinman’s concept of infraorders based on penis characters because it indicates the possibility of repeated evolution of penis lobe reduction during the phylogeny of the Dermaptera.

### Morphological Evolution vs. Molecular Evidence

The morphologies and life histories of the Arixeniidae and Hemimeridae are very different from those of the other Dermaptera. Adaptations to an epizoic life history resulted in the evolution of similar characters and thus the rise of homoplasies in data sets based on morphology.

The supraordinal classification of Grimaldi & Engel [14], which is based on paleontological material, comprises three suborders: the extinct Archidermaptera and Eodermaptera, and the recent Neodermaptera (corresponding to Hemimerina, Arixeniina, and Forficulina together in the traditional view). The internal phylogeny and classification of recent Dermaptera ( = Neodermaptera) has been in constant flux, with dramatically different arrangements of families and superfamilies by contemporaneous authors. Grimaldi & Engel [14] distinguish two infraorders within the Neodermaptera: the Protodermaptera and Epidermaptera. The Protodermaptera is basal including the families Karschielidae, Pygidicranidae, and Diplatyidae (sensu [19]), but may be paraphyletic, e.g., [9], [10]. Although the protodermapterans are well characterized by having ventral sclerites of equal size, carinae on the femora, and a segmented pygidium [10], [14], the validity of the infraordinal status of the Protodermaptera and the phylogeny within the infraorder must be verified by further analyses because the recent phylogenetic studies did not use representative material [11], [39], [42]. In particular, the phylogenetic position of the Karschielidae has not been studied by molecular methods. The Epidermaptera comprises the remainder of the recent families of earwigs. Based on his study of some morphological structures of the abdomen, Klass [12] reported that none of the abdominal characters contradicts a subordinate placement of *Hemimerus* within the Neodermaptera, and he noted weak support for a close relationship of *Hemimerus* to *Apachyus* (Apachyidae).

Haas [10] and Haas and Kukalova-Peck [9] performed extensive quantitative phylogenetic analysis of the Dermaptera based mainly on wing characters (especially the articulation and folding pattern of hind wings). Some of the characters seem to be apomorphic, but these characters cannot be used for the Hemimeridae and Arixeniidae because both of these epizoic groups have secondarily reduced wings. Recently, Tworzydlo et al. [14], [16]–[18] studied the ovary structure and initial stages of oogenesis of several dermapteran taxa, including the epizoic *Arixenia esau* Jordan, 1909. Their interpretation of the results supports the monophyly of the Dermaptera and the inclusion of the arixeniids in the Neodermaptera. Although the shared characters of the ovaries (elongated oviducts and a few, short ovarioles with a low number of follicles) support the position of the Arixeniina within the clade Forficuloidea comprising Chelisochidae+Forficulidae+Spongiphoridae, a placement of this taxon as a sister group of any of them is not strongly supported.

The families Chelisochidae and Forficulidae exhibit morphological autapomorphic characters on tarsomeres in that the third tarsal segment is inserted dorsally on the second tarsal segment and the second tarsal segment is enlarged [10]. The second tarsomere in the Forficulidae is lobed and very wide, while the second tarsomere in the Chelisochidae is not lobed but is enlarged into a peg-shaped process extending under the ventral surface of the third tarsomere [10], [11]. The third tarsal segment of both the Arixeniidae and Hemimeridae is inserted dorsally, which supports their relationship to the Chelisochidae and Forficulidae. The second tarsomere of the Hemimeridae is of the Forficulidae type (it is lobed and wide) and therefore could support the sister position of Hemimeridae+Forficulidae. The second tarsomere of the Arixeniidae is neither lobed nor enlarged but is a conspicuous process extending under the ventral surface of the third tarsomere. We cannot, however, exclude the possibility that the tarsomere characters in the Arixeniidae and Hemimeridae are adaptations to an epizoic life history rather than examples of synapomorphy.

Application of molecular phylogenetics has often generated hypotheses that are incongruent with results derived from a traditional approach based on morphological analyses [58], [59], [65]. Consequently, the “total evidence approach” may be complicated by conflicting signals between molecular and morphological data sets [66]. The phylogenetic reconstructions within a total evidence framework may be biased by the inclusion of phylogenetically misleading data, which are *a priori* more frequent in morphological data sets because of the greater potential for rapid adaptive change in particular phenotypic traits than in commonly used genetic traits. For example, the reduction of penis lobes, which was considered a synapomorphy in previous reconstructions of Dermaptera phylogeny [10], [12], [61]–[64], may result from parallel evolution, which is typical for reductive character states. From this point of view, molecular markers and corresponding tree-building methods are especially useful in phylogenetic resolution of groups with morphologically highly derived lineages because these methods effectively reveal relationships based on homologies [67], [68], as demonstrated in present study of epizoic lineages of earwigs.

## Supporting Information

Figure S1 (a) Bayesian phylogram of earwig families based on nuclear sequence data (18S and 28S ribosomal) and including only those parts that have sequence information for both *Arixenia* and *Hemimerus*. Numbers above branches indicate posterior probabilities. Figure S1 (b, c) Results of the exploration of MCMC convergence (of shortened alignment) using the AWTY (Are We There Yet?) approach. (b) Cumulative plot of the posterior probabilities of 20 splits at selected increments over one of two MCMC runs. (c) Comparative plot of posterior probabilities of all splits for paired one and two MCMC runs.
(TIF)
