# Supplementary figures and images for: When the Body Hides the Ancestry: Phylogeny of Morphologically Modified Epizoic Earwigs Based on Molecular Evidence
Source: PLoS One. 2013 Jun 24;8(6):e66900. doi: 10.1371/journal.pone.0066900 (PMC3691250; doi:10.1371/journal.pone.0066900)

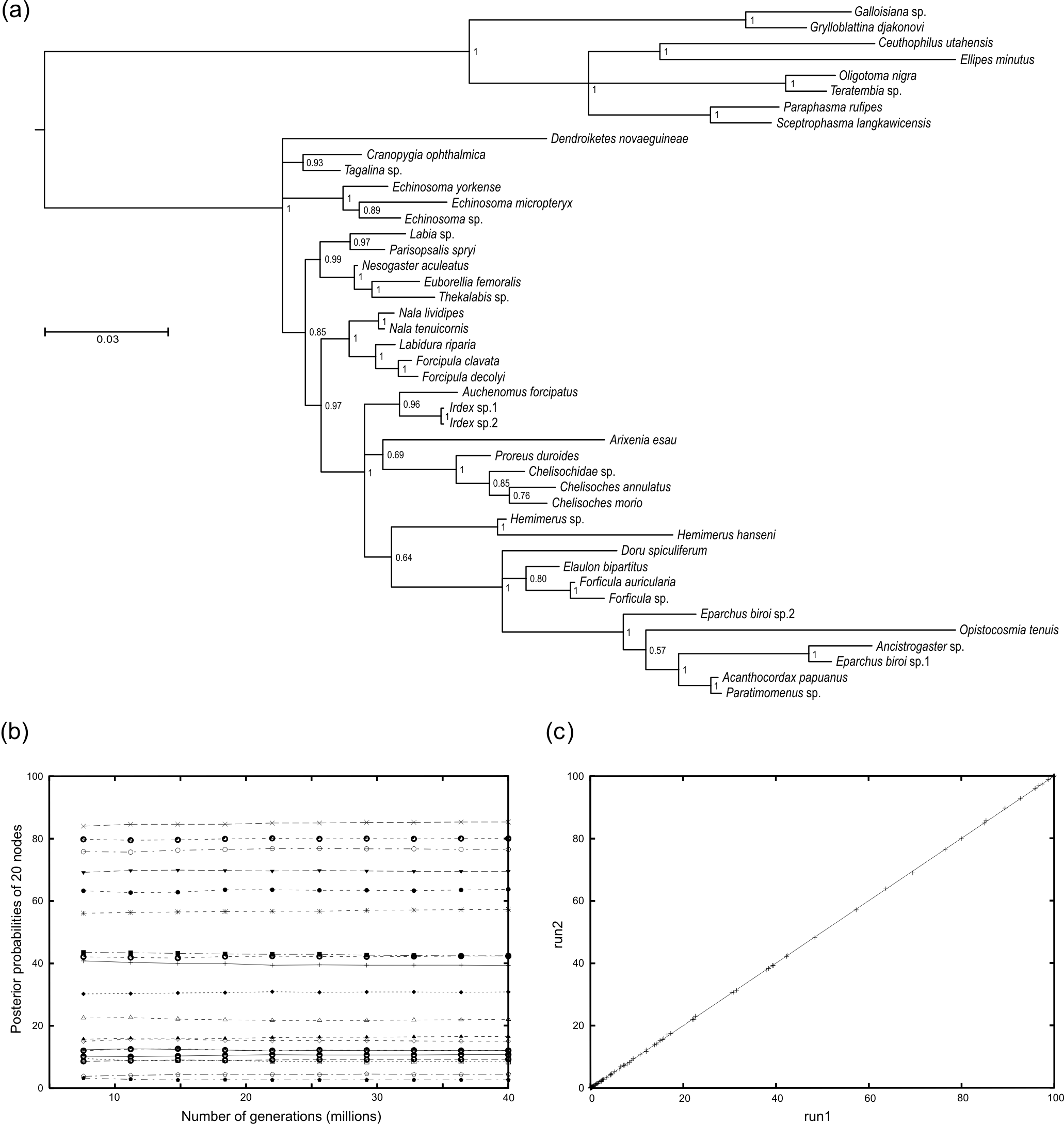

Supplement: Figure S1 — (a) Bayesian phylogram of earwig families based on nuclear sequence data (18S and 28S ribosomal) and including only those parts that have sequence information for both Arixenia and Hemimerus. Numbers above branches indicate posterior probabilities. Figure S1 (b, c) Results of the exploration of MCMC convergence (of shortened alignment) using the AWTY (Are We There Yet?) approach. (b) Cumulative plot of the posterior probabilities of 20 splits at selected increments over one of two MCMC runs. (c) Comparative plot of posterior probabilities of all splits for paired one and two MCMC runs. (TIF) [file pone.0066900.s001.tif]
